# Health-related quality of life and insulin resistance over a 10-year follow-up

**DOI:** 10.1038/s41598-021-03791-x

**Published:** 2021-12-21

**Authors:** Nijole Kazukauskiene, Aurelija Podlipskyte, Giedrius Varoneckas, Narseta Mickuviene

**Affiliations:** Laboratory of Behavioral Medicine, Neuroscience Institute, Lithuanian University of Health Sciences, Vyduno al. 4, 00135 Palanga, Lithuania

**Keywords:** Health care, Quality of life

## Abstract

The aim of the study was to investigate the association between insulin resistance (IR) and health-related quality of life (HRQoL) among citizens of Palanga in a 10-year follow-up. A randomized epidemiological study was performed with 835 subjects. The following data were examined using questionnaires: sociodemographic characteristics, behavioural factors, HRQoL and self-perceived health. Fasting blood samples were drawn from all participants, and biochemical tests were performed for glucose and insulin. IR was evaluated by the homeostasis model assessment of IR (HOMA-IR). In subjects with IR, after adjusting for various factors, logistic regression analysis showed that within 10 years, there was a significantly higher chance of deteriorating HRQoL in the areas of physical functioning (odds ratio [OR] = 1.15, p < 0.001), emotional role limitations (OR = 1.07, p = 0.034), social functioning (OR = 1.26, p = 0.004), pain (OR = 1.09, p = 0.005) and general health perception (OR = 1.07, p = 0.022). People with IR have a worse HRQoL, and as they age, they are significantly more likely to have a deterioration in their HRQoL than people without IR in the areas of physical functioning, emotional role limitations, social functioning, pain and general health perception.

## Introduction

Insulin, through its various effects on insulin target cells, plays an important role in regulating physiological blood glucose levels. Insulin resistance (IR) is a condition in which insulin-responsive cells do not respond to insulin as well as they should^1^. IR is one of the primary cause of metabolic syndrome^2–5^, which in turn reflects a real variety of closely linked risk factors. The aetiology of metabolic syndromes is complex and multifactorial. The key causes are obesity, physical inactivity, sedentary lifestyle and aging population^6–8^. The metabolic effects of IR, including hyperglycemia and dyslipidemia, appear to interact synergistically with increased blood pressure to promote vascular injury which can exacerbate the hypertension and the damage it causes to the cardiovascular system^9,10^.

IR occurs as part of a cluster of cardiovascular–metabolic abnormalities. A strong relationship exists between diabetes mellitus (DM) and cardiovascular disease. Cardiovascular disease is the most prevalent cause of mortality and morbidity in diabetic populations^11^. Given the seriousness of cardiovascular disease data, diabetes is becoming a greater burden^12^. IR precedes the development of diabetes, cardiovascular disease, and Alzheimer’s disease^13^. DM and impaired glucose tolerance have been associated not only with increased morbidity and mortality but also with poor quality of life^14,15^.

The assessment of IR indicators in everyday clinical practice is based on fasting glucose and insulin concentrations. The homeostasis model assessment of IR (HOMA-IR) is one of the most often used indirect indicators for characterizing this condition in the ‘steady-state’^16,17^. Using the HOMA-IR and the SF-36 health survey, cross-sectional data from 1212 participants in the Hertfordshire Cohort Study revealed that IR was associated with poor health-related quality of life (HRQoL) in domains of physical health but not in domains of mental health^18^.

IR was found be strongly correlated with an estimated measure of quality of life in patients with coronary heart disease but without known DM^19^.

HRQoL is a multidimensional term that relates to overall satisfaction with life and describes a patient's level of functioning across physical, emotional, and social domains. It represents the patient's perception of the impact of a disease and its treatment. Many variables influence HRQoL, including disease-related disability^20^.

To date, a correlation between IR and HRQoL has received little attention in the medical literature; therefore, it is still controversial and is not well understood. It is important to examine the association with worse HRQoL in this population. HRQoL reflects how an individual views and adapts to their symptom burden, mental distress, functional limitations, as well as how patients perceive their overall health. Thus, we aimed to investigate the association between HRQoL and IR among citizens of Palanga in a 10-year follow-up.

## Methods

### Study participants

We administered two surveys: the first was administered in 2003, and the second was administered in 2013. A random sample of 2500 citizens of Palanga aged 35–74 was recruited from the National Population Register in 2002. The citizens of Palanga were chosen because there was a close community with minor migration reflecting the population of the western part of Lithuania. The optimal size of the sample was calculated (1630 ± 33 individuals), ensuring representativeness of the population of Palanga aged 35–74 years. From the sample of 2500 citizens, 160 selected persons were not invited to participate in the study because they were not found at the given addresses, and 1602 persons (600 males and 1002 females) participated in the survey in 2003. The response rate for the first survey was calculated as follows: (1602/2340) × 100 = 68.5%. Ten years later, in 2013, the same people who participated in the first survey were invited to complete the second wave. In the period from 2003 to 2013, 158 persons (9.9%) who had participated in the first survey in 2003 died, 47 (2.9%) had changed their address, 20 (1.2%) declined to participate, 11 (0.7%) could not participate as a result of serious health problems, and 435 (27.2%) did not respond to the multiple invitations sent to them via mail. During the second survey, data from 931 people were collected, including 322 males and 609 females with ages ranging from 45–84 years. The first and second surveys were approved by the Ethics Committee for Biomedical Research at Lithuanian University of Health Sciences, Kaunas, Lithuania (Protocol code BE-2-25, approved 14 June 2012). Informed consent was obtained from all participants during both surveys. However, blood samples were collected from only 850 subjects, and fifteen (1.8%) subjects whose blood tests showed severe thyroid dysfunction were excluded from the analysis. The final study cohort consisted of 835 subjects, including 300 (35.9%) men and 535 (64.1%) women. The mean age of the study subjects was 63.5 ± 10.3 years. We analysed the data of both surveys’ participants (N = 835) and did not include those who participated in only one survey. The original source of method descriptions is described elsewhere^21^.

### Study procedure

The following data were assessed via questionnaires: sociodemographic characteristics (i.e., age, sex, height, weight, education and marital status), behavioural factors, HRQoL and self-perceived health. Fasting blood samples were drawn from all participants, and biochemical tests were performed for glucose and insulin. IR was calculated according to the formula HOMA-IR (HOMA-IR = (fasting plasma insulin [μIU/ml]) × (fasting plasma glucose [mmol/l])/22.5); normal rate of HOMA = ≤ 2.7.

### Measures

#### WHO-5 wellbeing test

The WHO-5 Well-being Index^22^ consisted of 5 questions reflecting the well-being of a person during the last 2 weeks: I feel cheerful and in good spirits; I feel calm and relaxed; I feel active and vigorous; I wake up feeling fresh and rested; my daily life is filled with things that interest me. The raw score is calculated by totalling the scores of the five items. The raw score ranges from 0 to 25; 0 represents the worst possible quality of life, and 25 represents the best possible quality of life. To obtain a standardized percentage score ranging from 0 to 100, the raw score is multiplied by 4. A standardized score of 0 represents the worst possible quality of life, whereas a score of 100 represents the best possible quality of life. Cronbach α = 0.876. The respondents who scored 50 or more were classified as not having depressive moods. Respondents who scored less than 50 were classified as having depressive moods, and they were considered to have an increased risk of depression.

#### Questionnaire on general data, behavioural factors and self-perceived health

The questionnaire on general data^23^ was used to collect information about the marital status, education, employment and income of respondents. The questionnaire assessing behavioural factors^23^ consisted of questions about smoking, alcohol consumption, and physical activity during the last year. The self-perceived health questionnaire^23^ consisted of questions about complaints and diagnosed diseases, medications used during the last year, frequency of stress events, and visits to any doctor.

#### Objective investigation

Arterial blood pressure (mmHg) was measured twice with a quicksilver sphygmomanometer (Riester 660/306, DIPLOMAT Presameter, Germany) on the right hand while a person was sitting, with a precision of 2 mm according to the methodological guidelines^24^. The average of two measurements was used for the analysis. If the participants' systolic blood pressure was greater than 140 mmHg and/or their diastolic arterial blood pressure was greater than 90 mmHg in the previous two weeks, they were considered hypertensive.

Body height was measured in stocking feet (without shoes) with a medical height rod. Body weight was measured without shoes using a medical scale (SECA 778, SECA Corporation, Hamburg, Germany) with a stadiometer. Body mass index (BMI) was calculated according to the following formula: BMI = body mass (kg)/height (m)^2^ using the height and weight measurement data. Overweight was diagnosed when BMI was 25.0–29.9 kg/m^2^, and obesity was diagnosed when BMI was 30.0 kg/m^2^ or more.

#### 36-Item Short Form Medical Outcome Questionnaire (SF-36)

The 36-item Short Form Medical Outcome Questionnaire (SF-36) consists of 8 multi-item subscales that assess HRQoL on 8 domains: physical functioning, social functioning, role limitations due to physical problems, role limitations due to emotional problems, mental health, energy/vitality, pain, and general health perception. Each of the 8 SF-36 domains is scored on scales from 0 to 100, with higher scores indicating better HRQoL^25^. The internal reliability (ɑ coefficients) of the 8 subscales was found to range between 0.71 and 0.85.

### Statistical analysis

The clinical and sociodemographic characteristics were reported by frequencies and percentages for the categorical variables and with means and standard deviations for the continuous variables. The variable distribution of similarity to normal was assessed visually and using the Kolmogorov–Smirnov and Shapiro–Wilk tests. Qualitative data were compared between groups without IR and with IR using Fisher’s χ^2^ test. Quantitative data were compared using the parametric two-tailed Student *t* test test or the nonparametric Mann–Whitney *U* test. Logistic regression analysis using an enter method was used to investigate whether the 10-year follow-up period (time), sex, age, and IR were related to different areas of quality of life (Model 1) and additionally adjusted for family status, education, employment, self-perceived health, frequent stressful events, depressed mood, alcohol use, smoking, illness during the past 12 months, and obesity (Model 2). Statistical analyses were performed with the Statistical Package for the Science Software v.22 (SPSS, Chicago, IL). The level of significance was set at p < 0.05.

### Ethical approval

All procedures in our study were approved by the Ethics Committee for Biomedical Research at Lithuanian University of Health Sciences, Kaunas, Lithuania (protocol code BE-2-25 and 14 June 2012 of approval) and conformed to the principles outlined in the Declaration of Helsinki.

### Informed consent

Informed consent was obtained from all individual participants included in the study.

## Results

### Baseline characteristics

Table 1 lists the participants’ sociodemographic characteristics of all participants stratified into groups without IR (HOMA-IR ≤ 2.7) and with IR (HOMA-IR > 2.7) in the survey at the 2003- and 2013-year follow-ups. As demonstrated in Table 1, significant differences in age, sex, education, BMI and self-related health were found between the study groups, both in the first survey in 2003 and the same in the second survey in 2013. Participants with IR were more likely to be older, male, have higher BMI, have lower levels of education, and have moderate self-related health. However, there were significantly fewer smokers and higher levels of physical activity in both groups after 10 years. In addition, systolic and diastolic arterial blood pressure was greater in participants with IR.

**Table 1:** The sociodemographic characteristics of subject groups without and with insulin resistance in the survey in 2003 and 2013.

Baseline characteristics	2003			2013			2003:2013	2003:2013
	Without IR group	With IR group	p-value	Without IR group	With IR group	p-value	Without IR group	With IR group
	n = 557	n = 278		n = 557	n = 278			
Age, years; mean ± SD	53.1 ± 10.6	54.9 ± 9.9	**0.016**	63.0 ± 10.5	64.8 ± 9.7	**0.015**	**< 0.001**	**< 0.001**
**Sex; n (%)**			**0.003**			**0.003**	–	–
Male	181 (32.5)	119 (42.8)		181 (32.5)	119 (42.8)			
Female	376 (67.5)	159 (57.2)		376 (67.5)	159 (57.2)			
BMI, kg/m^2^; mean ± SD	26.0 ± 4.3	30.0 ± 4.5	**< 0.001**	26.6 ± 4.1	31.0 ± 4.7	**0.002**	**0.013**	**0.005**
**Marital status; n (%)**			0.932			0.533	**0.004**	**0.007**
Married	420 (75.4)	211 (75.9)		376 (67.5)	181 (65.1)			
Alone	137 (24.6)	67 (24.1)		181 (32.5)	97 (34.9)			
**Education; n (%)**			**0.001**			**0.001**	0.287	0.839
Less than higher	369 (66.2)	217 (78.1)		351 (63.0)	214 (77.0)			
Higher	188 (33.8)	61 (21.9)		206 (37.0)	64 (23.0)			
**Employment; n (%)**			0.281			0.142	**< 0.001**	**< 0.001**
Employed	369(66.2)	173 (62.2)		277 (49.7)	123 (44.2)			
No employed	188 (33.8)	105 (38.8)		280 (50.3)	155 (55.8)			
**Self-rated health**			**0.014**			**0.018**	0.083	0.277
Good health	178 (32.1)	63 (22.7)		203 (36.4)	76 (27.3)			
Moderate	344 (62.0)	192 (69.1)		334 (60.0)	186 (66.9)			
Poor health	33 (5.9)	23 (8.3)		20 (3.6)	16 (5.8)			
Smoking regular; n (%)	198 (35.5)	112 (40.3)	0.182	85 (15.3)	38 (13.7)	0.541	**< 0.001**	**< 0.001**
Physical activity; n (%)							< 0.001	< 0.001
Daily walking in minutes > 60 min	206 (37.0)	93 (33.6)	0.316	412 (73.1)	184 (64.8)	0.015		
SBP, mm Hg; mean ± SD	120 ± 17.0	127 ± 16.0	**< 0.001**	128 ± 12.9	132 ± 12.5	**< 0.001**	**< 0.001**	**< 0.001**
DBP, mm Hg; mean ± SD	81.9 ± 7.2	86.68 ± 9.4	**< 0.001**	74.0 ± 7.2	75.7 ± 7.4	**0.002**	**< 0.001**	**< 0.001**

*BMI* body mass index, *IR* insulin resistance, *SBP* systolic blood pressure, *DBP* diastolic blood pressure; p-value of probability for comparison between groups (bolded numbers indicate significant differences, **p < 0.05**); data presented as n (%), mean ± SD.

### Comparison of health-related quality of life scores according to the presence of IR in the survey in 2003 and 2013

The HRQoL scores of the SF-36 questionnaire of the study (2003 and 2013) population according to the presence of IR are shown in Table 2. HRQoL according to the physical functioning subscale was significantly lower in the first study and after 10 years in subjects with IR than in subjects without IR. Additionally, after 10 years, HRQoL in the physical function subscale decreased significantly in both study groups according to the presence of IR, but the decrease in HRQoL was statistically significantly greater in the IR group. Physical role limitations subscale scores did not differ between groups in the first study, but 10 years later, the difference was significant. Significantly lower physical role limitations were found in IR individuals, although this dimension of HRQoL decreased significantly with age in both groups. Social function subscale scores were the same in both the IR and without IR groups in the first study; these scores did not change after 10 years in the group without IR and decreased significantly in the IR group, so the difference in the social function dimension of HRQoL was significantly worse in subjects with IR.

**Table 2:** Health-related quality of life scores of subject groups without and with insulin resistance in the survey in 2003 and 2013.

SF-36 domain scores, mean (95% CI)	2003			2013			2003:2013	2003:2013
	Without IR	With IR	p-value	Without IR	With IR	p-value	Without IR	With IR
	n = 557	n = 278		n = 557	n = 278			
Physical functioning	78.5 (77.1–79.9)	75.5 (73.6–77.3)	**0.009**	75.4 (73.6–77.1)	65.4 (62.5–68.3)	**< 0.001**	**< 0.001**	**< 0.001**
Physical role limitations	66.9 (64.2–69.5)	64.9 (61.3–68.4)	0.388	60.5 (57.3–63.7)	53.2 (48.4–58.0)	**0.014**	**< 0.001**	**< 0.001**
Emotional role limitations	67.4 (64.7–70.1)	69.0 (61.3–68.4)	0.480	66.6 (63.4–69.9)	64.8 (60.0–69.5)	0.515	0.687	0.125
Social functioning	75.4 (73.9–77.0)	75.4 (73.3–77.6)	0.998	76.5 (74.6–78.4)	69.7 (66.8–72.7)	**< 0.001**	0.304	**0.001**
Mental health	64.4 (63.0–65.8)	66.3 (64.7–67.9)	0.079	68.6 (67.1–70.0)	68.4 (66.–70.5)	0.911	**< 0.001**	**0.005**
Energy vitality	60.3 (58.9–61.7)	61.1 (59.4–62.8)	0.477	61.5 (60.0–63.0)	60.0 (57.8–62.2)	0.254	0.147	0.351
Pain	68.7 67.1–70.4)	67.8 (65.6–70.1)	0.516	69.3 (67.3–71.2)	65.2 (62.3–68.1)	**0.019**	0.636	0.093
General health perception	50.0 (48.8–51.2)	49.3 (47.9–50.8)	0.504	52.5 (51.1–53.9)	49.2 (47.2–51.1)	**0.007**	**< 0.001**	0.860

*IR* insulin resistance p-value of probability for comparison between groups (bolded numbers indicate significant differences, **p < 0.05**).

Scores on the mental health subscale increased significantly with age in both groups, but we did not find a significant difference in the groups according to the presence of IR. Scores on the pain subscale did not significantly change with age in either group, but after 10 years, the difference in HRQoL between groups became statistically significantly different, with worse HRQoL being observed in the IR group. No differences were found in the energy vitality and emotional role limitations subscales when evaluating the data in the groups according to IR over 10 years. The general health perception dimension of HRQoL did not change with age in the IR group; in the group without IR, it improved significantly, and after 10 years, the differences between the groups became significant.

In the IR group, the HRQoL became significantly worse than that in the group without IR.

### The change in health-related quality of life at the 10-year follow-up

Table 3 lists the participants’ change in SF-36 subscale scores between study groups in the period from 2003 until 2013. As demonstrated in Table 3, significant differences in the SF-36 subscales of physical functioning, social functioning and general health perception were found between the IR groups. Participants with IR had a significant decrease in the domains of the SF-36 subscales of physical functioning, social functioning and general health perception, showing their worse HRQoL in the 10-year period.

**Table 3:** Change in health-related quality of life scores of subject groups without and with insulin resistance in the period from 2003 until 2013.

SF-36 domain scores, mean (95% CI)	Without IR	With IR	p-value
ΔPhysical functioning	–3.1 (–4.7 to –1.6)	–10.1 (–12.9 to –7.2)	**< 0.001**
ΔPhysical role limitations	–6.4 (–9.8 to –2.9)	–11.6 (–17.0 to –6.3)	0.092
ΔEmotional role limitations	–0.75 (–4.4 to 2.9)	–4.3 (–9.7 to 1.2)	0.283
ΔSocial functioning	1.1 (–0.98 to 3.1)	–5.7 (–8.9 to –2.5)	**0.001**
ΔMental health	4.2 (2.6 to 5.8)	2.1 (–0.04 to 4.3)	0.132
ΔEnergy vitality	1.2 (–0.43 to 2.9)	–1.1 (–3.5 to 1.2)	0.110
ΔPain	0.51 (1.6 to 2.6)	–2.6 (–2.5 to –0.01)	0.098
ΔGeneral health perception	2.5 (1.2 to 3.8)	–0.19 (–2.3 to 1.9)	**0.033**

*IR* insulin resistance, *SF-36* The 36-item Short Form Medical Outcome Questionnaire; p-value of probability for comparison between groups (bolded numbers indicate significant differences, **p < 0.05**).

### Logistic regression models

Year, sex, age and IR were included in Model 1 as possible candidates for factors having a significant association with HRQoL area deterioration after 10 years. Logistic regression analysis revealed that the 10-year period had a significantly higher chance of deteriorating HRQoL in the following areas: physical functioning (OR 1.65, p < 0.001), physical role limitations (OR 1.56, p < 0.001), emotional role limitations (OR 1.43, p = 0.002) and social functioning (OR 1.40, p = 0.003). Female gender had a positive impact on physical functioning (OR 1.29, p = 0.018), physical role limitations (OR 1.32, p = 0.010), social functioning (OR 1.55, p < 0.001), pain (OR 1.45, p = 0.005) and general health perception (OR 1.30, p = 0.140). Age was not significantly positively associated with only mental health and general health perception. IR was significantly and independently associated with higher odds for decreases in the physical functioning (OR 1.36, p = 0.005), social functioning (OR 1.70, p < 0.001), mental health (OR 1.32, p = 0.008), energy vitality (OR 1.40, p = 0.002), pain (OR 1.28, p = 0.018) and general health perception (OR 1.40, p = 0.002) dimensions of HRQoL.

After adjusting for year, sex, age, IR, marital status, education, employment, self-perceived health, frequent stressful events, depressed mood, alcohol use, smoking, illness during the past 12 months, and body mass index, subjects with IR had a significantly higher chance of deteriorating HRQoL in the dimensions of physical functioning (OR 1.15, p < 0.001), emotional role limitations (OR 1.07, p = 0.034), social functioning (OR 1.26, p = 0.004), pain (OR 1.09, p = 0.005) and general health perception (OR 1.07, p = 0.022) after a 10-year period, as seen in Model 2 (Table 4).

**Table 4:** Results from regression analyses in which year, sex, age, and insulin resistance are regressed on SF-36 subscales.

Independent variable	Model 1 OR (95% CI)		p-value	Model 2 OR (95% CI)		p-value
**Physical functioning**						
Time: 2013(1)/2003(2)	1.65	(1.32–2.06)	** < 0.001**	1.64	(1.30–2.08)	** < 0.001**
Sex: Males (1)/Females (2)	1.29	(1.04–1.59)	**0.018**	1.22	(0.96–1.54)	0.104
Age	1.04	(1.04–1.06)	** < 0.001**	1.04	(1.02–1.05)	** < 0.001**
IR without (1)/with (2)	1.36	(1.10–1.68)	**0.005**	1.15	(1.07–1.23)	** < 0.001**
**Physical role limitations**						
Time: 2013(1)/2003(2)	1.56	(1.25–1.95)	** < 0.001**	1.53	(1.20–1.94)	** < 0.001**
Sex: Males (1)/Females (2)	1.32	(1.07–1.63)	**0.010**	1.15	(0.91–1.46)	**0.246**
Age	1.05	(1.04–1.06)	** < 0.001**	1.04	(1.02–1.05)	** < 0.001**
IR without (1)/with (2)	1.17	(0.95–1.45)	0.150	1.04	(0.82–1.44)	0.253
**Emotional role limitations**						
Time: 2013(1)/2003(2)	1.43	(1.14–1.79)	**0.002**	1.47	(1.15–1.87)	**0.001**
Sex: Males (1)/Females (2)	1.09	(0.88–1.45)	0.427	1.07	(0.84–1.36)	0.600
Age	1.04	(1.03–1.05)	** < 0.001**	1.03	(1.02–1.05)	** < 0.001**
IR without (1)/with (2)	1.11	(0.90–1.38)	0.332	1.07	(1.01–1.38)	**0.034**
**Social functioning**						
Time: 2013(1)/2003(2)	1.40	(1.37–2.10)	**0.003**	1.43	(1.13–1.82)	** < 0.001**
Sex: Males (1)/Females (2)	1.55	(1.26–1.92)	** < 0.001**	1.47	(1.16–1.87)	**0.002**
Age	1.04	(1.03–1.05)	** < 0.001**	1.03	(1.02–1.05)	** < 0.001**
IR without (1)/with (2)	1.70	(1.37–2.10)	** < 0.001**	1.26	(1.03–1.17)	**0.004**
**Mental health**						
Time: 2013(1)/2003(2)	0.95	(0.76–1.18)	0.629	0.98	(0.78–1.23)	0.933
Sex: Males (1)/Females (2)	1.09	(0.89–1.34)	0.408	1.03	(0.82–1.29)	0.822
Age	0.99	(0.99–1.00)	0.262	1.00	(0.98–1.01)	0.711
IR without (1)/with (2)	1.32	(1.07–1.63)	**0.008**	1.06	(0.99–1.23)	0.054
**Energy vitality**						
Time: 2013(1)/2003(2)	1.13	(0.91–1.40)	0.273	1.12	(0.97–1.53)	0.107
Sex: Males (1)/Females (2)	1.11	(0.91–1.36)	0.316	0.97	(0.77–1.22)	0.801
Age	1.01	(1.00–1.02)	**0.011**	1.01	(0.99–1.02)	0.386
IR without (1)/with (2)	1.40	(1.14–1.71)	**0.002**	1.03	(0.97–1.09)	0.370
**Pain**						
Time: 2013(1)/2003(2)	1.14	(0.92–1.42)	0.223	1.09	(0.97–1.57)	0.101
Sex: Males (1)/Females (2)	1.45	(1.18–1.78)	** < 0.001**	1.43	(1.13–1.80)	**0.003**
Age	1.01	(1.00–1.02)	**0.005**	1.01	(0.99–1.02)	0.327
IR without (1)/with (2)	1.28	(1.04–1.58)	**0.018**	1.09	(1.03–1.16)	**0.005**
**General health perception**						
Time: 2013(1)/2003(2)	1.08	(0.87–1.35)	0.465	1.04	(0.81–1.30)	0.297
Sex: Males (1)/Females (2)	1.30	(1.06–1.60)	**0.014**	1.21	(0.96–1.53)	0.106
Age	1.01	(0.99–1.02)	0.090	1.00	(0.98–1.01)	0.580
IR without (1)/with (2)	1.40	(1.13–1.72)	**0.002**	1.07	(1.01–1.14)	**0.022**

*IR* insulin resistance, *OR* odds ratio, *CI* confidence interval.

Model 1—Adjusted for year, sex, age, insulin resistance.

Model 2—Adjusted for year, sex, age, insulin resistance, marital status, education, employment, self-perceived health, frequent stressful events, depressed mood, alcohol use, smoking, illness during past 12 months, body mass index. Bolded numbers indicate significant **p < 0.05.**

## Discussion

This longitudinal study regarding HRQoL among individuals in the western part of Lithuania indicated that decreases in HRQoL were significantly related to IR, as evaluated by HOMA-IR. The major finding in the present study is that subjects both with IR and without IR reported lower SF-36 scores in the physical functioning and physical role limitation dimensions after 10 years. However, the SF-36 subscale of mental health scores increased significantly with age in both groups, indicating better HRQoL. Over the course of 10 years, significant changes were observed in domains of the SF-36 subscales of physical functioning, social functioning and general health perception, showing worse HRQoL in subjects with IR. Furthermore, the results of this study suggest that future research should continue to examine people who have IR. It is a cause for concern that within 10 years, subjects with IR have a significantly higher chance of deteriorating HRQoL in the areas of the five of the eight subscales: physical functioning, emotional role limitations, social functioning, pain and general health perception.

To our knowledge, our study is the first to assess the association between IR and HRQoL over a 10-year follow-up. IR is an important factor that needs to be investigated for to precisely identify which dimensions of HRQoL are more affected by the presence of IR. Based on Schlotz et al.’s findings, IR and its related measures are associated with poor HRQoL in the domains of physical health but not in domains of mental health^18^. According to Sayer et al., glucose intolerance or an elevated plasma glucose level may induce poor muscle control and hinder physical activity^26^, so it is likely that IR affects physical disability. Better mental health status thus leads to more physical activity, which in return has a positive association with better mental health and physical health^27^. Furthermore, better past mental health is found to reduce cigarette consumption, which then has a positive effect on present health^28^. In addition, the study by Ohrnberger et al.^29^ implies that health investments may be considered intervention channels through which mental and physical health can be improved at old age.

According to the findings of Eddy et al.^30^, people who acquire IR have a roughly threefold higher risk of coronary artery disease than those who do not. In participants with IR, normalizing IR lowered cardiovascular risk by approximately 55%. The study by Agewall et al. showed that an estimated measure of quality of life was significantly and independently associated with IR in patients with coronary heart disease and without known DM^19^.

According to growing data, IR is thought to have a role in the pathophysiology of cognitive impairment and neurodegeneration. Insulin has a significant impact on cognitive performance^31^. Poorer HRQoL is an independent predictor of cognitive function, as established in the recent research of community-based older persons^32^.

Together, the data from these studies and our data suggest that IR seems to be one of the most important factors determining HRQoL in healthy people. This is a potentially modifiable situation that could be changed with dietary and lifestyle improvements. As a result, we suggest that more research on HRQoL is needed, especially in the area of sexuality^33^, to support and investigate the health problems related to ageing and HRQoL improvements.

There is a growing body of evidence that shows a connection between metabolic syndrome and deterioration in HRQoL^6,34–36^. Unfortunately, there is still little research examining IR as the primary cause of metabolic syndrome in association with HRQoL.

The present study aimed to draw attention to the effects that IR can have on HRQoL, as IR is still an important public health issue that can accompany a variety of risk factors (Supplementary Information).

## Conclusions

The results of this study suggest that people with IR have a worse HRQoL, and as they age, they are significantly more likely to have deterioration in their HRQoL compared to people without IR in the areas of physical functioning, emotional role limitations, social functioning, pain and general health perception after a 10-year period.

## Supplementary Information

Supplementary Information.

## Data Availability

The datasets analysed during the current study are available from the corresponding author upon request.

## References

[1] Shanik MH (2008). Insulin resistance and hyperinsulinemia: Is hyperinsulinemia the cart or the horse?. Diabetes Care.

[2] Freeman, A. M. & Pennings, N. in *StatPearls* (StatPearls Publishing Copyright © 2021 (StatPearls Publishing LLC, 2021).

[3] Beale EG (2013). Insulin signaling and insulin resistance. J. Investig. Med..

[4] Sesti G (2006). Pathophysiology of insulin resistance. Best Pract. Res. Clin. Endocrinol. Metab..

[5] Perry RJ, Samuel VT, Petersen KF, Shulman GI (2014). The role of hepatic lipids in hepatic insulin resistance and type 2 diabetes. Nature.

[6] Tziallas D (2012). The impact of the metabolic syndrome on health-related quality of life: A cross-sectional study in Greece. Eur. J. Cardiovasc. Nurs..

[7] Kim K, Park SM (2018). Association of muscle mass and fat mass with insulin resistance and the prevalence of metabolic syndrome in Korean adults: A cross-sectional study. Sci. Rep..

[8] Han TS (2013). Quality of life in adults with congenital adrenal hyperplasia relates to glucocorticoid treatment, adiposity and insulin resistance: United Kingdom Congenital adrenal Hyperplasia Adult Study Executive (CaHASE). Eur. J. Endocrinol..

[9] da Silva AA (2020). Role of hyperinsulinemia and insulin resistance in hypertension: Metabolic syndrome revisited. Can. J. Cardiol..

[10] Mirr M, Skrypnik D, Bogdański P, Owecki M (2021). Newly proposed insulin resistance indexes called TyG-NC and TyG-NHtR show efficacy in diagnosing the metabolic syndrome. J. Endocrinol. Investig..

[11] Matheus AS (2013). Impact of diabetes on cardiovascular disease: An update. Int. J. Hypertens..

[12] Wild S, Roglic G, Green A, Sicree R, King H (2004). Global prevalence of diabetes: Estimates for the year 2000 and projections for 2030. Diabetes Care.

[13] Onyango AN (2018). Cellular stresses and stress responses in the pathogenesis of insulin resistance. Oxid. Med. Cell. Longev..

[14] Alberti KG, Zimmet P, Shaw J (2006). Metabolic syndrome—A new world-wide definition. A Consensus Statement from the International Diabetes Federation. Diabet. Med..

[15] Noh JW, Chang Y (2019). Self-rated health and the risk of incident type 2 diabetes mellitus: A cohort study. Sci. Rep..

[16] Wallace TM, Levy JC, Matthews DR (2004). Use and abuse of HOMA modeling. Diabetes Care.

[17] Menik Hettihewa, D. L., Palangasinghe, D. S., Jayasinghe, D. S., Gunasekara, D. S. & Weerarathna, D. T. Vol. 5 (eds Dr Srinivas Kakkilaya Bevinje & Dr Shatharam Baliga B) (Dr. B.S. Kakkilaya, 2006).

[18] Schlotz W (2007). Specific associations of insulin resistance with impaired health-related quality of life in the Hertfordshire Cohort Study. Qual. Life Res..

[19] Agewall S, Henareh L (2008). Quality of life and insulin resistance in patients with coronary heart disease. Coron. Artery Dis..

[20] Erceg P (2013). Health-related quality of life in elderly patients hospitalized with chronic heart failure. Clin. Interv. Aging.

[21] Andruškienė, J., Podlipskytė, A., Martinkėnas, A. & Varoneckas, G. Risk factoRs and heaRt Rate vaRiability as the pRedictoRs of caRdiovasculaR death: Ten yeaR health outcomes community-based study. *Redaktorių taryba*, 81 (2014).

[22] WHO. World Health Organization: Regional Office for Europe Well-Being measures in primary health care: The DepCare Project. Consensus meeting, Stockholm (1998).

[23] Grabauskas, V., Petkevičienė, J., Šakalytė, E., Kriaučionienė, V. & Veryga, A. Suaugusių Lietuvos žmonių gyvensenos tyrimas, 2011 = Health Behaviour among Lithuanian Adult Population, 2011. *Kaunas LSMU* (2011).

[24] Pickering TG (2005). Recommendations for blood pressure measurement in humans and experimental animals: Part 1: Blood pressure measurement in humans: A statement for professionals from the Subcommittee of Professional and Public Education of the American Heart Association Council on High Blood Pressure Research. Circulation.

[25] Ware JE, Sherbourne CD (1992). The MOS 36-item short-form health survey (SF-36). I. Conceptual framework and item selection. Med. Care.

[26] Sayer AA (2005). Type 2 diabetes, muscle strength, and impaired physical function: The tip of the iceberg?. Diabetes Care.

[27] Gerber M, Pühse U (2009). Review article: Do exercise and fitness protect against stress-induced health complaints? A review of the literature. Scand. J. Public Health.

[28] Szatkowski L, McNeill A (2015). Diverging trends in smoking behaviors according to mental health status. Nicotine Tobacco Res..

[29] Ohrnberger J, Fichera E, Sutton M (2017). The relationship between physical and mental health: A mediation analysis. Soc. Sci. Med..

[30] Eddy D, Schlessinger L, Kahn R, Peskin B, Schiebinger R (2009). Relationship of insulin resistance and related metabolic variables to coronary artery disease: A mathematical analysis. Diabetes Care.

[31] Ma L, Wang J, Li Y (2015). Insulin resistance and cognitive dysfunction. Clin. Chim. Acta.

[32] Ezzati A (2019). Health-related quality of life, cognitive performance, and incident dementia in a community-based elderly cohort. Alzheimer Dis. Assoc. Disord..

[33] Llaneza P (2009). Insulin resistance and health-related quality of life in postmenopausal women. Fertil Steril.

[34] Saboya PP (2016). Metabolic syndrome and quality of life: A systematic review. Rev. Lat. Am. Enfermagem..

[35] Deihim T (2015). Which insulin resistance-based definition of metabolic syndrome has superior diagnostic value in detection of poor health-related quality of life? Cross-sectional findings from Tehran Lipid and Glucose Study. Health Qual. Life Outcomes.

[36] Cadeddu C, Nocco S (2016). Effects of metformin and exercise training alone or in combination, on cardiac function in individuals with insulin resistance. Cardiol. Ther..

