# Supplementary material for: Health-related quality of life and insulin resistance over a 10-year follow-up
Source: Sci Rep. 2021 Dec 21;11:24294. doi: 10.1038/s41598-021-03791-x (PMC8692503; doi:10.1038/s41598-021-03791-x)
Supplement: Supplementary file 1 — Supplementary Information. [file 41598_2021_3791_MOESM1_ESM.pdf]

GENERAL DATA QUESTIONNAIRE

Attention: After the reading all the answers, choose the one that is most acceptable to you and circle the number next to the option you have chosen. Please, answer very detail the questions presented without standard statements.

1. **Code** __________________
2. **Date** day-month-year ___________________
3. **Sex**

   Female ☐

   Male ☐
4. **Date of birth** ___________________
5. **Family status**

   Married/living with someone ☐

   Single ☐

   Divorced ☐

   Widowed ☐
6. **Education**

   Primary ☐

   Not finished secondary ☐

   Secondary/College ☐

   University ☐
7. **What kind of work do you do for most of the year?**

   Agricultural and forestry work ☐

   Industry, buiding etc. ☐

   Student ☐

   Housewife ☐

   Retired ☐

   Unemployed ☐

   Office, mental job, service sector ☐

   Other ☐
8. **Work regime:**

   Only daytime ☐

   Daytime/night time (Shift work) ☐
9. **Average income per family member/month?** ______________

SELF-EVALUATED HEALTH QUESTIONNAIRE

Attention: After the reading all the answers, choose the one that is most acceptble to you and circle the number next to the option you have chosen. Please, answer very detail the questions presented without standard statements.

1. How do you assess the current health?

Good ☐ Rather good ☐ Fair ☐ Rather poor ☐ Poor ☐

2. How many times do you attend the doctor (In-hospital, out-patient department, except odontologist) during the last 12 months? _________

If no visits, mark the Ă0ñ.

3. Have you experienced stress in the last month?

No at all ☐

Yes, a little, but I donćt usually experience it ☐

Yes, but other persons experience more than usually ☐

Yes, my life is just unbearable ☐

4. Have you experienced following pain during last month at night:

<u>Attention:</u> Please mark in each row of the table + prie ĂYesñ arba ĂNoñ. No rows in the table can be left blank.

	**Pain**	**Yes**	**No**
1.	Headache		
2.	Stomach ache		
3.	Toothache		
4.	Joint pain		
5.	Diabetic foot pain		
6.	Back pain		
7.	Lower back pain		
8.	Muscle ache		
9.	Other pain		

5. Which of these diseases have you been diagnosed with and treated in the last 12 months?

<u>Attention:</u> Please mark in each row of the table + prie ĂYesñ arba ĂNoñ. No rows in the table can be left blank.

	**Diseases**	**Yes**	**No**
1.	Arterial hypertension		
2.	Diabetes mellitus		
3.	Myocardial infarction (Heart attac)		
4.	Angina pectoris		
5.	Coronary artery disease		
6.	Rheumatic arthritis		
7.	Spinal diseases		
8.	Emphysema (pulmonary enlargement)		
9.	Chronic bronchitis or asthma		
10.	Chronic pyelonephritis		
11.	Others		

6. Have you taken any medication in the last month:

<u>Attention:</u> Please mark in each row of the table + prie ĂYesñ arba ĂNoñ. No rows in the table can be left blank.

	**Medicines**	**Yes**	**No**
1.	Sedatives		
2.	Sleeping pills		
3.	Antialergics		
4.	Antidepressants		
5.	Pain-relieving medicines		
6.	Antihypertensives		
7.	Antiarrythmics		
8.	Anticholesterol drugs		
9.	Cough-reducing medicines		
10.	Others		

LIFESTYLE ASSESSMENT QUESTIONNARE

Smoking

1. Are you currently smoking?

Yes, regularly ☐ Somethimes ☐ No ☐

If ñNoò, please, continue to answer the questions in the next section ĂAlcoholñ.

2. How many cigarettes, papiroses or pipes you smoke per day on average?

1ï 5 ☐; 6ï 14 ☐; 15ï 25. ☐ > 25 ☐

Alcohol

1. How often do you drink alcohol (including beer)?

Daily ☐; 2-3 times per week ☐; Once per week ☐; 2-3 times per month ☐

Several times per year ☐; Donót use it at all ☐.

Physical activity during the last year

1. How many minutes during a normal work day do you sit (If you are not working ï usually during the day)?

_______________ minutes

2. How many minutes a day do you walk?

______________minutes
